# Binge Drinking and Problem Gambling Association in Adolescents and Young Adults

**DOI:** 10.3389/fnhum.2022.845505

**Published:** 2022-05-13

**Authors:** Laura Angioletti, Michela Balconi

**Affiliations:** ^1^International Research Center for Cognitive Applied Neuroscience (IrcCAN), Università Cattolica del Sacro Cuore, Milan, Italy; ^2^Research Unit in Affective and Social Neuroscience, Department of Psychology, Università Cattolica del Sacro Cuore, Milan, Italy

**Keywords:** adolescence, binge drinking, gambling, executive functions, decision-making

## Introducing the Link Between Binge Drinking (BD) and Problem Gambling

While the association between alcohol use disorders and problem gambling has been object of discussion in previous research literature, less is known about the link between Binge Drinking (BD) and risk gambling in adolescents.

Starting from a definition of those two phenomena, shared definitions and criteria for BD (Maurage et al., 2020) and for problem gambling (Neal et al., 2005) have been proposed. Nonetheless, such a topic is still a matter of debate. In the literature concerning BD, one of the most recognized definition of the phenomenon is the one of the National Institute on Alcohol Abuse and Alcoholism (NIAAA, USA), which describes it as the consumption of >56 g (women) or >70 g (men) of ethanol in <2 hours, bringing blood alcohol concentration to at least 0.08%. As for the relatively recent proposal of an integrated definition of BD, see Maurage et al. (2020). At-risk/problem gambling can, instead, be described as a behavior characterized by “difficulties in limiting money and/or time spent on gambling, which leads to adverse consequences for the gambler, his/her relatives, or the community” (pp. 1) and tends to encompass gamblers who have experienced problem gambling without meeting the diagnostic criteria of gambling disorder (Neal et al., 2005).

Interestingly, in terms of prevalence, evidence has shown that both BD (Kraus and Nociar, 2016; Substance Abuse Mental Health Services Administration, 2018) and gambling (Shaffer and Hall, 2001; Calado and Griffiths, 2016; Calado et al., 2017) show higher prevalence rates in adolescents and young adults than adults. Despite gambling on lottery products (e.g., scratch tickets), private card games (e.g., poker), putting bets on games of skill, and sports betting are the most regularly reported behaviors for gambling in teenagers in most North American, European, and Australasian studies (Delfabbro et al., 2016), the increased prevalence rates of gambling in this age group may be explained by the augmented availability of gambling opportunities via the internet, mobile phone and interactive television and by the normalization of this behavior in society (Calado et al., 2017).

Previous research suggested the association between BD and problem gambling. For instance, college students who matched the criterion for BD were more likely to engage in sports betting, video and regular poker, the web, office pools, and other skill games than those who did not satisfy the criteria (Bhullar et al., 2012). In US college athletes, problem gambling showed the strongest association with at-least-weekly heavy episodic drinking, followed by marijuana and cigarette use (Huang et al., 2011). Sundqvist et al. (2015) examined the association between BD and at-risk gambling in the general Swedish population and found they are linked behaviors. However, age and smoking had the greatest impact on the association, suggesting the relevance of demographic variables for this population. Even socio-demographic factors, such as the housing conditions (e.g., not living with both parents in the same household and not having a university-educated parent) were associated with both heavy episodic drinking and risk gambling (Kaltenegger et al., 2019). The association between BD and problem gambling in adolescents has been confirmed also by a cross-sectional study involving thirty-three European countries (Gori et al., 2015; Molinaro et al., 2018).

Although socio-demographic factors seem to mediate the relationship between BD and problem gambling, it is necessary to underline that even factors related to cerebral and cognitive development are involved in the development of this co-occurrence of risky behaviors.

This contribution follows a specific structure. We will start by describing the neurodevelopmental reasons why adolescents are at risk for BD and gambling behavior. Then, we will focus on how cognitive factors also contribute to this association and we will discuss decision-making dysfunction in adolescents with BD as the common ground with problem gambling. With the aim of providing not only an overview of the impaired brain functions that come into play in this comorbidity (i.e., the *pars destruens*), the fourth section focuses on the *pars construens*, that is the description of potential clinical interventions at the intersection between BD and problem gambling.

In conclusion, the need for tailor-made prevention and treatment for adolescence with BD and problem gambling so to address these multiple risk domains will be stressed.

## Why Teenagers Are at Risk for BD and Gambling

Firstly, what are the neurodevelopmental reasons why teenagers are at risk for BD and gambling behaviors? To date, the investigation of the cognitive and neurological basis of adolescents' conduct has yielded substantial advancements in the neurodevelopmental field (Ernst et al., 2006, 2009; Steinberg, 2010; Casey et al., 2011). Notably, the typical development of pubertal age includes the development of brain structures that support the consequent cognitive development, encompassing the ability to process rewards and punishments but also self-awareness and self-regulation processes, which allow managing the propensity to novelty-seeking and risk-taking that is typical of adolescent behavior. And therefore, where does the aspect of vulnerability to risk behaviors typical of this age fit in?

Previous neurobiological models described how subcortical neural circuitries develop faster than cortical regions (*i.e.*, prefrontal cortex) in adolescents (Casey et al., 2011). The misalignment relative to the maturation of neural areas that support the increase in motivation and satisfaction of needs and structures that deal with motivation regulation makes the adolescent's necessary adjustment to societal norms and individual ambitions complex. A peculiarity of typical adolescent development concerns the importance of the short-term rewards that derive from one's choices and conducts, which tends to be greater than the capacity for self-regulation. This aspect exposes them to situations that can be risky for their health, but also to new necessary challenges for the typical human development (Windle et al., 2008).

Four different theoretical models in cognitive neuroscience described adolescence neurocognitive mechanisms related to the development of regulated behavior. According to the model of the “dual system” (Steinberg, 2010) and the theory of “maturational imbalance” (Casey et al., 2011), higher risk-taking throughout adolescence leads to a mix of augmented sensitivity to gains and primal impulse control. Also, the “triadic model” stated that adolescence is marked by variations in the interplay between approach behavior, avoidance tendencies, and regulatory systems (Ernst et al., 2006, 2009). Indeed, the approach system typically appears hypersensitive, whereas the avoidance system is somewhat hyposensitive. Also, the development of the regulation system of Executive Functions (EFs) may not yet be sufficiently mature to control and adaptively modulate the other two systems. Finally, a neuroeconomic approach to teenage decision-making (van Duijvenvoorde and Crone, 2013) try to account for the association between risky choice, sensitivity to gains and losses, and social perspective-taking typically characterizing the adolescence developmental phase. Specifically, the immaturity of cortical areas supporting the “hot” executive system (*i.e.*, the system including functions activated under motivationally significant and affective conditions, such as the ability to delay gratification and affective decision-making; Zelazo and Carlson, 2012) may explain the reason why the explicit knowledge about the risks and outcomes of the conduct, which depends on a developed cognitive control, seems to be in place, while the affective control of the choice is not (Steinberg, 2005), thus leading to poor decision-making processes.

With reference to these cognitive and executive factors in the context of addiction in adolescents, Noël (2014) focused specifically on the individual differences in cognition and neural functions accounting for the onset of alcohol use disorder and gambling in adolescence. It addressed the vulnerable points and discussed the role of dysfunctional EF and decision-making in this population. However, the impairment of those abilities has been detected even in association with BD in adolescents and this is what will be discussed in the next section. From a lifespan-oriented perspective, it is essential to conduct neurodevelopment studies exploring the neurocognitive vulnerability to risky behaviors in adolescents. In fact, adolescence and youth are periods characterized by significant structural and functional changes in the brain. Implementing such dysfunctional behaviors (BD and gambling) during that critical period of growth becomes potentially predictive of future brain, cognitive, behavioral, and psychological alterations, which can manifest themselves through functional consequences over time.

## Executive and Decision-Making Dysfunction in Adolescents With BD: Common Ground With Problem Gambling

Both neural and behavioral impairments related to EF have been noted in adolescents with alcohol use disorder and gambling (Noël, 2014), but also in young binge drinkers (Lannoy et al., 2019). Indeed, adolescents and young patients with BD display an alteration of cognitive functioning supported by the cortico-frontal regions and hippocampus (Squeglia et al., 2012). More specifically, adolescents and young adults showing BD and heavy-drinking have a thinner and lower volumes of gray matter in prefrontal cortex and cerebellar regions, as well as attenuated white matter development. When presented with working memory, language learning, and inhibitory control tasks, they also demonstrate increased brain activity in fronto-parietal areas. Binge and heavy drinkers demonstrate enhanced brain response to alcohol signals in mesocorticolimbic areas such as the striatum, anterior cingulate cortex, hippocampus, and amygdala, compared to controls or light drinkers (Cservenka and Brumback, 2017).

Also, besides EF impairments, several studies showed that binge drinkers had poorer decision-making abilities measured with the Iowa Gambling Task (Goudriaan et al., 2007, 2011; Xiao et al., 2009, 2013; Moreno et al., 2012).

At the neural level, different neurocognitive findings have been described depending on the decision-making process assessed through the decision-making task (Cservenka and Brumback, 2017). In BD individuals, riskier decision-making behavior was linked to dorsal striatum hypoactivation (Jones et al., 2016). Binge drinkers, on the other hand, showed increased activity in the prefrontal, orbitofrontal, and upper parietal dorsolateral cortex in association with hazardous choices in a risk-taking task (Worbe et al., 2014). Xiao et al. (2013) findings at the Iowa Gambling Task showed that, compared to never drinkers, binge drinkers display an augmented bilateral activation of the insula and left amygdala, two affective neural hubs linked, respectively to the translation of inner body information into feelings and the elaboration of reward and emotions. Taken all together, these findings suggest poor decision-making ability in BD both at the behavioral and neural level, with a potential social implication for the individual, that is the higher vulnerability of adolescents to other risky behaviors such as alcohol use disorder or problem gambling (Xiao et al., 2013).

Of interest for this contribution, here we intend to discuss and propose how this decision-making dysfunction could be one of the main common grounds between BD and problem gambling. This assumption is supported by the findings focusing on decision-making in adolescents with gambling (*e.g.*, Ciccarelli et al., 2016; Cosenza et al., 2017) and adolescents with BD. For example, Goudriaan et al. (2007) found that poor binge drinkers' decision-making abilities at the Iowa Gambling Task were unrelated to their impulsivity score. Again, Moreno et al. (2012) found decision-making deficits in the absence of inhibitory control impairments. Also, Na et al. (2019) assessed, by means of Event-Related Potentials, the ability to use feedback for decision-making in female college students binge drinkers and found a deficit in the early evaluation of positive vs. negative feedback, concluding that this deficit may impact on dysfunctional behavioral decision-making.

These findings support Verdejo-Garcia's (2017) hypothesis that, while decision-making abilities and executive processes may be linked, individuals can also have a dissociated pattern characterized by decision-making impairments but preserved EF (as in the case of Moreno et al., 2012), or functional decision-making but EFs impairment (Balconi and Angioletti, 2021; Balconi and Campanella, 2021) highlighting the process independent contribution (Verdejo-Garcia, 2017). For example, in our recent work on internet use vulnerability in healthy young adults (a different population than the one discussed in this opinion article), we have highlighted how high scores in the internet addiction test seem to correspond to an attentional bias for internet addiction-related cues (pictures representing online gambling), while decision-making abilities appeared preserved (Balconi and Angioletti, 2021). This example is consistent because it refers to a different population, potentially preclinical and in which decision-making is preserved. It has been reported here in order to further underline the possible dissociation between impaired executive functioning and decision-making.

At the level of theoretical understanding, this possible dissociation has yet to be deepened and systematized in relation to adolescents with BD and problem gambling. This reflection could be a useful starting point to deepen this aspect also in the entire field of “new” behavioral addictions. Additionally, it proves useful to identify a dissociation between effectively impaired functions in this population to develop tailored preventive or rehabilitative approaches. This knowledge may have clinical implications ranging from the combination of multiple training approaches to precision medicine. For instance, multiple training approaches could combine psychosocial interventions with specific cognitive training on decision-making dedicated to adolescents with BD and problem gambling.

With the aim of providing applications and highlighting the first practical implications of this contribution, the next section aims to focus on the clinical interventions at the intersection between BD and problem gambling, with reference to adolescents and young age groups where possible.

## Clinical Interventions at the Intersection Between BD and Problem Gambling

As discussed up to this point, the adolescent phase consists of a stage of life in which the individual is particularly exposed to transgressions and risky impulses, such as alcohol consumption, substance use (Charrier et al., 2020), and gambling behavior (Noël, 2014). The proof related to the co-occurrence of risky behaviors in adolescents may have consequences for designing approaches and planning preventive and treatment clinical interventions: the most promising intervention programs to reduce risk behaviors seems to be those that target multiple risk domains (Spring et al., 2012).

With reference to the available clinical interventions at the intersection between BD and problem gambling in adolescents, Martin et al. (2020) reviewed the effectiveness of therapeutic interventions for adolescents using alcohol and/or other drugs in the Australian context and found a paucity of quality research on this topic. Such, a critical issue seems primarily due to the fact that the studies that are accessible do not account for all the treatment strategies actually adopted in the field. While some of these interventions have a solid evidence base, others (such as encounter groups and journaling) require further in-depth research before being used with teenagers (Martin et al., 2020). In general, psychosocial, rather than pharmacological, interventions are recommended as first-line treatment for adults with BD (Rolland and Naassila, 2017). However, well-defined protocols and tested clinical recommendations would be necessary to work both in terms of prevention and clinical intervention with these vulnerable samples.

Despite the paucity of systematic reviews on this topic, this contribution wants to underline the need for clinical interventions dedicated to cognitive factors, such as the neurocognitive aspects of EFs, in adolescents with BD and problem gambling. To date, there is a lack of neurocognitive interventions specifically dedicated to EF and decision-making for this population. Specific training and tools to prevent and rehabilitate the EF processes impaired in BD, particularly inhibitory control (Lannoy et al., 2019), have been addressed before. Also, neuromodulation techniques (specifically, transcranial Direct Current Stimulation over the left dorsolateral prefrontal cortex; Den Uyl et al., 2015) have been recognized as interesting avenues for targeting inhibitory control in this population.

Nonetheless, when there is a co-occurrence of problem gambling in adolescents, the clinical interventions might benefit from the integration of specific training on working memory, self-regulation (Noël, 2014), and decision-making. Given the shortage of research exploring intervention on combined BD and problem gambling, one possibility is to refer to the interventions available to date for the treatment of neurocognitive aspects related to problem gambling in adolescence. Frisone et al. (2020) underlined that the data referred to the treatment of gambling in adolescence appeared as limited as the studies proposing preventive approaches. Typically, psychological treatment approaches adopted with adults with gambling disorder–such as motivational interviewing, cognitive behavioral therapy, and brief mindfulness interventions (Menchon et al., 2018)–are also proposed for adolescents with problem gambling. These interventions may have an impact on decision-making capabilities with problem gambling (*i.e.*, cognitive behavioral treatment; Oldershaw et al., 2012). Also, cognitive training (Luquiens et al., 2019) and neuromodulation could be new avenues for enhancing EF in gambling. For instance, neuromodulation applied over the dorsolateral prefrontal cortex has been associated to enhanced decision making and cognitive flexibility in a sample of male adults with gambling disorder (Soyata et al., 2019).

Although there is some evidence that psychological intervention programs can improve EF and decision-making in teenagers with BD and problem gambling, novel neurocognitive approaches are required to specifically target the impaired decision-making abilities and immature self-regulation system in this population. In addition, even though research and clinical practice are still in an embryonic stage for these approaches, other types of intervention (such as non-invasive neurofeedback) could be warranted to investigate if neuromodulation interventions are promising even at this stage of life and for this behavioral co-occurrence.

## Concluding Remarks

To summarize, little is known about the link between BD and problem gambling in adolescence. However, while socio-demographic factors appear to influence this relationship, even factors related to adolescent cerebral and cognitive development should be taken into consideration, since they constitute a factor that makes teenagers vulnerable to the development of this risky behavior pattern. A first consideration is that more studies in this area are needed to better understand the co-occurrence or possible causality between BD and gambling in adolescents and young adults. These future studies will have to take into account that there are aspects related to the age of prevalent manifestation of problem gambling (14–15 years) compared to BD (20–21 or 18–25 years; Barnes et al., 2009; Substance Abuse Mental Health Services Administration, 2018), as well as to personality traits related to these two risky behaviors that could constitute barriers to conducting studies in this field and that must be taken into account to obtain a complete picture of this co-occurrence.

The impairment of EF and decision-making in adolescents could be considered a critical common feature of both BD and problem gambling and this aspect could deserve further attention in this field. Despite several studies assessing potential cognitive impairment in young BD and (to a lesser extent) problem gamblers, no studies have explored the cognitive performance of individuals with these two risk behaviors. This constitutes a gap in the literature that could be filled in the next few years and before developing adequate neurocognitive interventions for this population. Indeed, one of the more practical goals of this discussion is to solicit the development of tailor-made preventive and treatment interventions for adolescence with BD and problem gambling, in order to address these multiple risk domains, as well as EFs (especially decision-making) efficiency.

Therefore, the additional value of this contribution consists of (i) the specific focus on the EFs and decision-making in adolescents showing the comorbidity between BD and problem gambling; (ii) the distinction between a possible impairment in decision-making while EFs are preserved in this population, (iii) highlighting the lack of neurocognitive interventions specifically dedicated to EFs and decision-making for this population with BD and problem gambling. Despite its novel characteristics, one of potential shortcomings of this work is that it is an opinion article only providing comments on the interpretation of recent data in the research area of BD and problem gambling in teenagers, rather than a thorough assessment of the literature.

Future research directions should address these limitations and conduct studies that delve deeper into the link between these two behaviors, as well as into the potential neuro-functional and cognitive alterations associated with them, with particular attention to executive functions and decision-making. These studies could then consequently form the basis for the research and development of new preventive and rehabilitative neurocognitive treatments, including for example cognitive training and neuromodulation techniques, specifically dedicated to EF and decision-making in this population.

## Author Contributions

LA and MB wrote the first draft and each section of the manuscript and contributed to the manuscript final writing and revision, read, and approved the submitted version.

## Conflict of Interest

The authors declare that the research was conducted in the absence of any commercial or financial relationships that could be construed as a potential conflict of interest.

## Publisher's Note

All claims expressed in this article are solely those of the authors and do not necessarily represent those of their affiliated organizations, or those of the publisher, the editors and the reviewers. Any product that may be evaluated in this article, or claim that may be made by its manufacturer, is not guaranteed or endorsed by the publisher.
